# Smartphone application for adolescents with anorexia nervosa: an initial acceptability and user experience evaluation

**DOI:** 10.1186/s12888-021-03478-7

**Published:** 2021-09-25

**Authors:** Benjamin Naccache, Laure Mesquida, Jean-Philippe Raynaud, Alexis Revet

**Affiliations:** 1 Service Universitaire de Psychiatrie de l’Enfant et de l’Adolescent, CHU de Toulouse, Hôpital Purpan, Place du Dr Baylac, TSA 40031, 31059 Toulouse cedex 9, France; 2 CERPOP, Université de Toulouse, Inserm, UPS, Toulouse, France; 3 CIC 1436, Team PEPSS « Pharmacologie En Population cohorteS et biobanqueS », Toulouse University Hospital, Toulouse, France

**Keywords:** Anorexia nervosa, Eating disorders, Smartphone, Mobile applications, mHealth, Focus groups, User centered design, Qualitative research, Adolescent

## Abstract

**Background:**

Anorexia Nervosa (AN) is a key target for E-Health programs considering the many barriers hindering patients’ access to care and the disorder’s severity. Although these programs have become more common and effective, they often have low adherence, especially among youth. This can hinder their implementation and effectiveness in real-world settings. User experience partly overlaps with the acceptability field and may provide insight into factors affecting adherence and adoption of E-Health programs. This study aimed to explore early acceptability and user experience of a companion app prototype for adolescents with AN using user-centered design methods.

**Methods:**

We developed a prototype containing self-help material and emotions and behaviors evaluation and management features. Then we conducted a mixed evaluation combining semi structured focus group interviews and questionnaires in a clinician group and an AN patient group. We analyzed data using thematic analysis and descriptive statistics.

**Results:**

The app’s overall appeal was adequate. The user experience questionnaire revealed the weakest dimensions, including novelty, dependability, and efficiency versus stimulation (i.e., ability to induce motivation to use the product) and perspicuity (i.e., easy to understand, to get familiar with). The qualitative data analysis revealed three central axes: acceptability, features, and use. We identified acceptability barriers and facilitators such as the importance of design and customization, especially for adolescents. Psychoeducation was a major feature for participants, as patients highlighted the difficulties they encountered when seeking disorders-related information.

**Conclusions:**

This study shows the importance of including users in the different stages of an e-health intervention development, in order to identify their needs, general use and compliance patterns, to improve adherence and adoption of the program and its effectiveness.

## Introduction

Anorexia nervosa (AN) has one of the highest premature mortality rates among psychiatric disorders [1–3]. It may lead to multiple psychiatric and somatic complications [4] and have a significant impact on quality of life [5]. Despite the severity of this disorder and while many studies have highlighted the importance of early diagnosis and interventions to improve recovery prospects [6, 7], there are many barriers that may delay AN’s diagnosis and treatments. These barriers include the lack of training and resources of primary care professionals about eating disorders (EDs) or limited access to EDs services because of geographical barriers, long waiting lists and rigid admission rules [8, 9]. Moreover, the impact of COVID-19 has exacerbated these problems of access to care. Indeed, pandemic-related restrictions induced many disruptions in usual care conditions, such as face-to-face therapies, combined with an increase in EDs-related demands for care [10–12].

In this context, the widespread distribution of smartphones, and thus the day-to-day interventions and assessment opportunities they provide through applications, makes them relevant devices in improving patients’ care [13]. Digital interventions have proven their effectiveness in treating psychiatric disorders [14–16] and several studies have shown the effectiveness of online E-Health ED programs in decreasing ED behaviors, attitudes and beliefs [17]. Their effects appear to be based on enhancing motivation to change [18, 19] and increasing patients’ commitment within therapy [20]. Furthermore, these interventions seem to be cost-effective ways of increasing the accessibility and availability of mental health care services for individuals with ED symptoms [21, 22].

Even though online E-Health ED programs become more common, recent reviews showed that smartphone applications targeting ED, especially AN, are still rare and poorly evaluated [17, 23–25]. They also highlighted that existing applications failed to incorporate all smartphone capabilities that could deliver an entirely personalized intervention. Aiming to determine which components of web-based self-help interventions are associated with ED symptoms improvement, Barakat et al. [17] found that using different media channels is a beneficial feature of the interventions, while automated feedbacks (i.e., reminder, personalized message, summary of self-monitoring input data, etc.) appear to be associated with less improvement. Conversely, some authors showed that personalized feedbacks are prone to enhance digital interventions’ effectiveness [26, 27]. Smartphones apps allow a more flexible and personalized use of feedbacks, rendering this type of digital intervention efficient and easy to commit to. These are important features to consider while developing E-health programs to guarantee the best user experience possible. Digitally copying content from a manual is not enough to create engaging interventions, and E-health programs often have a high drop-out rate [15–17, 22, 28, 29], especially among children and adolescents [30], which undermines their effectiveness. Taking into account that there is an ever-growing number of apps available on the market, adopting a new one and using it over time becomes a challenge. One out of four mobile apps is never used once installed and 26% of all apps are discarded after a single use [31, 32]. Factors contributing to the adoption of an e-health intervention also need to be taken into account from the development’s very beginning [30] and user experience seems to play a major part in whether a product is adopted or not [33]. A product’s adoption, being the first step in users’ commitment to it, is necessary if we hope to spread its use on a larger scale, which is one of the great benefits of using new technologies.

To build a functional product, an app’s creation process requires several important steps before programming. The first stage is the identification and definition of the theoretical frame [34]. This step aims to precisely build the app’s functionalities, based on a sturdy theoretical model, and relying on empirical data, which is essential to create an efficient final product [27, 35]. In the second step, evaluating user experience during a short period of time, simulating the app’s first use, allows to modify the program before developing it further based on users’ expectations, to facilitate its adoption in real-life conditions [15, 36, 37]. Qualitative evaluation is a relevant tool in user-centred design allowing to better approach users’ experience [34, 38].

By following these two steps, we first developed an AN companionship app prototype for teenagers. Then, we conducted a questionnaire and qualitative study in a group of expert practitioners and a group of patients suffering from AN, aiming to evaluate the app’s early acceptability as well as users’ needs and experience.

## Method

### Prototype development

#### Unguided self-help program

**Fig. 1:**
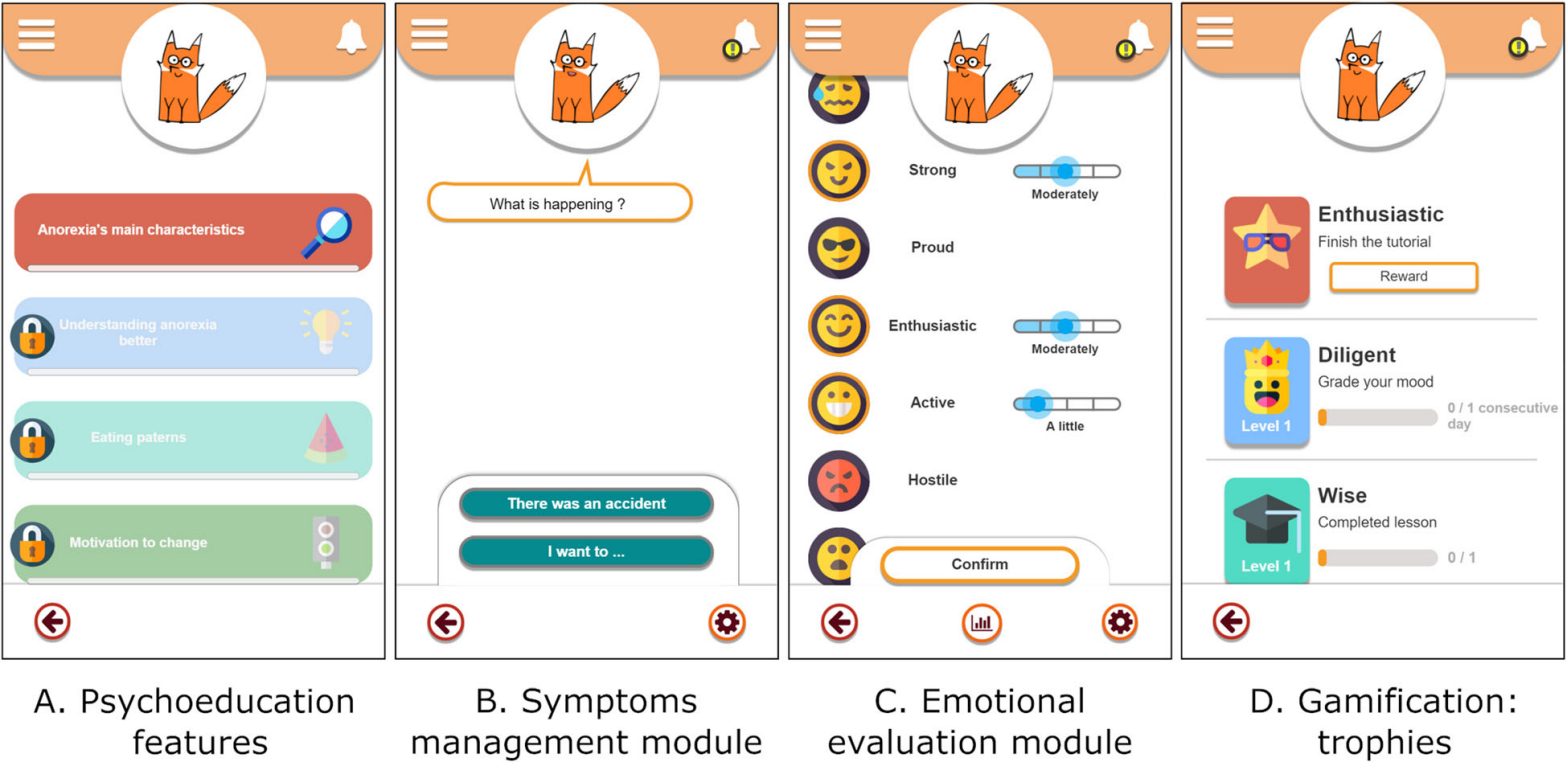
Design and features of the application prototype made using Axure RP9 Software (9.0.0.3727). Picture (**A**) shows the psychoeducational module. Picture (**B**) shows the symptoms management module. Picture (**C**) shows the emotional evaluation module. Picture (**D**) shows the gamification system using trophies. Icon made by Freepik from www.flaticon.com

The app would offer an unguided self-help program combined with psychoeducation features used in cognitive-behavioral therapy (CBT) and motivational interview strategies. Although self-help programs in AN are still scarce compared to other EDs, some studies have suggested that they could be relevant tools in enhancing usual “face-to-face” treatment’s efficiency, or in preventing AN relapse [39, 40]. Moreover, using motivational strategies could also improve this type of program’s efficiency in increasing motivation to change [41] (Fig. 1).

#### Emotional management program

The app would offer a guide in evaluating and managing negative emotions and behaviors focused on weight loss. It is based on several models that showed that emotional dysregulations are linked to AN and play a role in creating and maintaining the disorder [42, 43]. In AN patients, emotional dysregulation is characterized by the lack of knowledge and strategies in managing efficiently and with flexibility negative affects, having difficulties in identifying precisely its emotions and using inadequate emotion management strategies. Several studies stressed the relevance of offering strategies aiming to minimize or tolerate emotions for those patients, as well as to encourage and help identify precisely the patient’s emotions [42–45]. This module would thus offer useful skills for emotion management, based on what is used in CBT, therapy that has proven to be efficient in treating AN [46, 47]. It would also rely on what is suggested in dialectical behavior therapy, for which recently developed programs [48], focusing on excessive emotional control, have shown to be effective in AN treatment [48, 49]. Emotional evaluation was done using a French translation of the *Positive and Negative Affect Schedule* (PANAS) [50, 51], which is an auto-evaluating questionnaire made of two 10-items scales enabling to measure positive and negative emotions. It has been modified to better fit the app’s functions.

#### Gamification elements

Gamification elements consist of the use of game design elements in non-game contexts [52, 53]. Many studies have highlighted the importance of gamification elements to offer engaging experience that improve user’s participation and motivation to change [52, 54, 55]. Although these strategies are being implemented in mental health fields to improve patients’ commitment and the intervention’s effectiveness, they still are seldom used in EDs [53]. Therefore, we have put forward different gamification elements in this prototype, such as feedback on achieved improvement, the possibility to win trophies when the patient uses the app, and the opportunity to personalize its companion by unlocking special content.

### Evaluation

#### Study population and enrolment

Enrollment was conducted in November 2020 and group interviews took place from December 2020 to January 2021. The study’s design required two groups: the first was made of seven practitioners with prior experience in ED treatment who were enrolled from Toulouse Teaching Hospital. The second group was made up of eight inpatients, aged 12 to 18, suffering from AN as defined by DSM-5 criteria. We aimed to have a homogeneous patient population with experience about psychiatric care. Patients with suicidal thoughts or major restrictive symptoms with denial of the disorder were excluded due to their overwhelming symptoms.

#### Evaluation procedure

The qualitative data were collected through semi-structured group interviews lasting an average of 100 min and taking place at the Toulouse University Hospital. The interviews were conducted by a child psychiatrist (LM) and a resident in psychiatry (BN). They were digitally recorded and then transcribed. Their main objective was to explore and encourage different perspectives on user experience offered by the prototype, using the impulses of group dynamics. The first part of the interview was a 10 to 20 min individual testing time, during which participants could explore the app prototype. The aim was to reproduce the user’s first experience at the time of the app’s installation. Afterwards, the participants completed a questionnaire evaluating user experience. Then, a discussion time was organized, during which the moderator guided the participants’ debate using questions previously defined in the study’s interview guide.

#### Population characteristics

Patients’ sociodemographic characteristics were collected through questionnaires distributed at the interview’s beginning. Additional data on disease, weight, height, smartphone ownership were collected from the patient population. For practitioners, data on profession and ED patients care experience were also collected.

#### User experience questionnaire

The User Experience Questionnaire (UEQ), created by Laugwitz, et al. [56, 57], is made of 26 items evaluating different user experience dimensions, namely attractiveness, the product’s instrumental aspects (using perspicuity, efficiency, and dependability sub-scales) and non-instrumental aspects (using stimulation and novelty sub-scales).

#### Qualitative data

User experience is a recent concept, defined as the combination of elements related to the way people use an interactive product. User experience design process enables to guarantee that the designer’s point of view matches the user’s ones [34]. The two main user experience research models have been developed by Hassenzahl’s [58] and Mahlke and Thüring’s [59]. According to these authors, the perceived quality of a system would be built from their instrumental and non-instrumental qualities perception. Instrumental qualities are all elements linked to what makes the product functional. They are related to usability notions (i.e., aspects concerning the interface) and perceived usefulness, as found in the acceptability field. Non-instrumental qualities are a product’s characteristics that go beyond technical functionalities, aiming to meet user’s needs and desires, to stimulate him. For Mahlke and Thüring [34, 60], these two elements bring about emotional reactions that will impact user experience. Thanks to smartphones-related characteristics, users can easily test a mobile app and immediately feel a certain level of satisfaction. According to several research models concerning the adoption of new technologies [32, 33], satisfaction is one of the main links between the intent to use and the continuous use of a mobile app. This satisfaction in itself proceeds from user experience [33, 34].

We used these models to create the interview’s questionnaire (Table 1).

**Table 1:** Semi-structured interview guide

Interview guide	Investigated notion
**Initiation question**	
*In general, what would you (your patients) expect from an app like this one?*	Needs
**Part 1: Instrumental data**	
*How would you (your patients) use this app in your (their) care?*	Use
*In what way does this app and its features seem easy or on the contrary difficult to grasp?*	Usability
**Part 2: Non-instrumental data**	
*According to you, what could be described as pleasant or unpleasant in using this app?*	Stimulation
*What difficulties could you (your patients) face in this app’s daily use?*	Engagement
**Part 3: Modifications**	
*If you had to suggest features modifications, additions, or removals in this app, what would they be?*	Acceptability Features needs
*What modifications, additions or removals could favor your (your patients’) daily use of this app?*	Engagement Acceptability

#### Data analysis

The data collected during focus groups were analyzed thematically using the NVivo 12 (12.6.0.959) software. The interviews were recorded on a dictaphone and were later transcribed verbatim. First, every transcription was read several times without undergoing thematization. Then, after focusing on the research’s objectives, the transcription underwent a continuous thematization, in light of these objectives, without any theme grouping. Each theme was designed to describe what was transcribed without interpretation or generalization. A themes list was constructed simultaneously. Afterwards, starting from the second transcription analysis, recurrences were grouped under one theme. Once all of the corpus had undergone thematization, themes were analyzed according to their focal, opposition, recurrence, or junction point. BN conducted the analyses and codes were then discussed during group meetings which both enriched the analysis and served as a quality control process. This allowed us to construct the major phenomena trends’ overview in the form of a thematic arborescence [61]. Although we achieved a degree of data redundancy, we did not reach data saturation. The methodological criteria were checked retrospectively according to the COREQ (Consolidated criteria for Reporting Qualitative research) checklist [62].

The quantitative data collected by the UEQ was described using means and confidence intervals (CIs) and compared to a benchmark determined by M. Schrepp et al. [57] using the analysis tools they provided.

## Results

### Participants

The patient group was comprised of eight individuals with a mean age of 15.5 years (standard deviation; SD = 1.07) (Table 2). They were all female inpatients suffering from AN with a mean BMI of 14.69 kg/m^2^ (SD = 1.78). Most of them were in high school (*n* = 7), one did not provide data about her education. All of them had smartphones. The clinician group was comprised of seven individuals (*n* = 4 females, *n* = 3 males) with a mean ED patient care experience of 6.64 years (SD = 5.09). There were three psychiatrists, one pediatrician, one nutritionist physician, one psycho-motor therapist and one nurse.

**Table 2:** Sociodemographic characteristics of patients (*n* = 8) suffering from anorexia nervosa and practitioners (*n* = 7) with prior experience in eating disorders treatment

Variables	Values
Patients	
Age, mean +/− sd	15.5 (+/− 1.07)
Female, n (%)	8 (100)
BMI, mean +/− sd	14.69 (+/− 1.78)
Adolescent’s level of education, n (%)	
High school	7 (87.5)
Did not respond	1 (12.5)
Smartphone possession, n (%)	8 (100)
Practitioners	
Age, mean +/− sd	36.71 (+/−7.38)
Female, n (%)	4 (57.14)
EDs patient care experience (years), mean +/− sd	6.64 (+/− 5.09)
Profession, n (%)	
Psychiatrist	3 (42.86)
Pediatrician	1 (14.29)
Nutritionist physician	1 (14.29)
Psycho-motor therapist	1 (14.29)
Nurse	1 (14.29)

*BMI* Body mass index, *Sd* Standard deviation

### Questionnaire

**Fig. 2:**
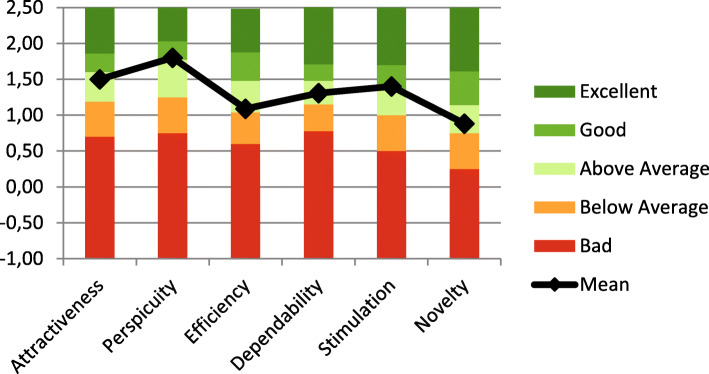
User Experience Questionnaire (UEQ) results compared to the UEQ benchmark. The UEQ offers a benchmark, which contains data from 452 product evaluations done with the UEQ (with a total of 20,190 participants). It is updated once a year. It classifies a product into five categories: Excellent: the result for the evaluated product is in the range of the 10% best results; Good: 10% of the results in the benchmark data set are better than the result for the evaluated product and 75% of the results are worse; Above average: 25% of the results in the benchmark are better and 50% of the results are worse; Below average: 50% of the results in the benchmark are better and 25% of the results are worse; Bad: the result for the evaluated product is in the range of the 25% worst results

The prototype’s overall first impression, measured by its attractiveness, was in the “above average” category set by the benchmark, with a score of 1.5 (CI = 1.022–1.978) (Fig. 2). Its non-instrumental aspect was measured using novelty and stimulation subscales, indicating scores of 0.88 (CI = 0.267–1.449) and 1.40 (CI = 0.890–1.910), respectively. Compared with the benchmark, this novelty score was defined as “above average”, even though the Inventive/Conventional and Creative/Dull items had the most negative critics. The stimulation score was “good”. The prototype’s instrumental aspect was measured using the following subscales: perspicuity, with a score of 1.80 (CI = 1.291–2.309), which is considered “good”; efficiency, with a score of 1.09 (CI = 0.607–1.571), considered as “above average”; dependability, with a score of 1.31 (CI = 0.917–1.695), considered as “above average”. The item with the most negative critics was Inefficient/Efficiency.

### Qualitative data

#### Acceptability

##### Theme: installation barrier and facilitators

**Fig. 3:**
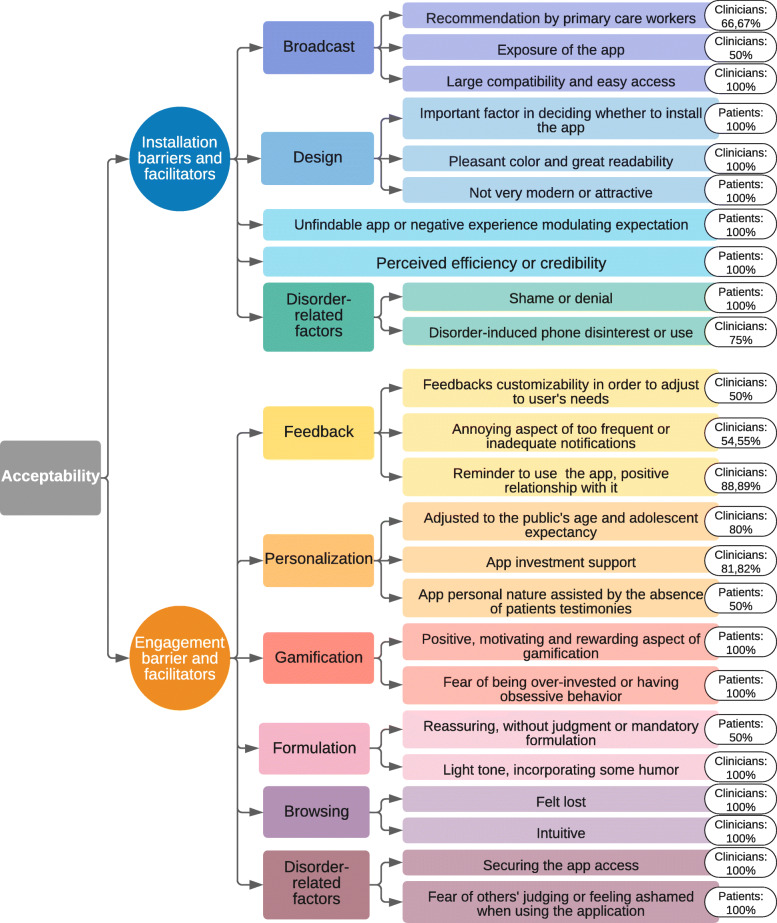
“Acceptability” axis thematic arborescence describing main themes and subthemes with percentage of the highest group’s coding rate per theme

For some patients, the app’s design and graphics were important in deciding whether to install the app, even though it did not matter as much afterwards (Fig. 3). Generally, whereas professionals were rather satisfied by the design, patients’ opinions were less positive. Some thought that it was not very modern or attractive. Both groups mentioned that external factors, like disorder-related ones or prejudices against the tool, could hinder its installation: disorder-induced phone disinterest, shame, denial, or disbelief in the app’s efficiency; whereas trusting the app’s data and believing in its usefulness could favor its installation. Participants insisted on their expectations that such a tool had to be well-known and recommended by primary care workers, favoring its installation. Clinicians talked about the importance of visibility, imagining an app with free access on all platforms, and easily found through search engines. Some patients said they had already tried to search for apps without finding one that suited them, thus creating expectations regarding this tool. For one patient these negative experiences could conversely cause reluctance in using the app.

##### Theme: engagement barrier and facilitators

For both group it turns out that feedback (i.e., sound alerts and notifications) could play a positive part in facilitating long-term commitment, by sending reminders to use the app, improving the user’s relationship with it, and simply by making it seem pleasant and encouraging. It seemed important that they were adjustable, specifically in their activation. Indeed, participants feared that if they became too frequent or invasive, they could endanger the user’s commitment. Both groups also worried that notifications could remind patients of their disorder. The app’s and the companion’s personalization features were perceived positively as an investment support and were expected. For patients and clinicians, the companion must be adapted to the target public’s age. If not, it could limit their commitment, especially in adolescents. Some participants felt that the app’s absence of testimonies was a positive feature, limiting the risk of comparing oneself with others and guaranteeing the app’s personal nature. Comments about gamification features were mixed. Some found them to be acceptable, “motivating” or even “gratifying”, whereas others were worried about trophies potentially inducing obsessive behaviors, with the risk of over-engaging to the point of cheating. Comments about browsing were also mixed in clinicians. Some found the app “simple to use” “intuitive”, whereas others felt “lost”. Among those who felt lost, some felt stimulated to explore and use the app, and recognized it as a positive aspect, while those who felt as though they did not control the app thought it could be “annoying”. Patients were comfortable browsing the app and reported no specific concern about it. Some participants worried that using the app could expose their disorders, especially when some features (like notifications) were activated. They feared that this could potentially increase the patient’s sense of shame surrounding the disorder, towards him/herself or others, endangering the commitment to the app. Meanwhile, some patients expected the app to feel “secure” “reassuring” and “not judging”. Finally, participants said that using mandatory formulation could impede commitment. Some professionals felt it was necessary to use a light tone and incorporate some humor.

#### Features

##### Theme: coping strategies

**Fig. 4:**
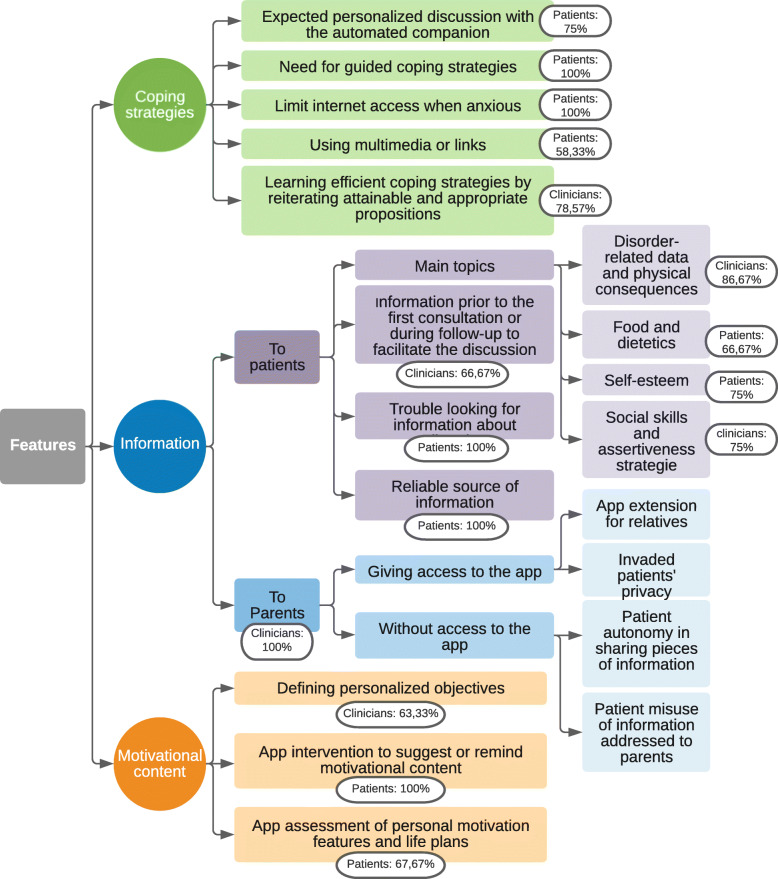
“Features” axis thematic arborescence describing main themes and subthemes with percentage of the highest group’s coding rate per theme

Most participants said they expected to be able to communicate and to be helped by the app through solutions suggestion when they felt overwhelmed by their symptoms (Fig. 4). Several patients said the proposed communication with the automated companion was acceptable, and expected a personalized discussion, needing to be “listened to” and “understood”. For one clinician the robot allowed a continuous presence. Some patients said that it was difficult for them to implement solutions when they were only suggested via words. They needed to be guided when they felt anxious, with, for example, audio tracks, music or links to videos that could be directly available through the app. They expected features that would allow them to shift their focus from their symptoms, using distractions, meditation, or relaxation apps, enabling them to open up to new experiences. Some even mentioned ways to limit their internet access, preventing them from encountering information that could exacerbate their symptoms. However, these expectations were nuanced by other patients, who declared preferring to keep the app’s features under control. For clinicians, this tool was useful in helping to find solutions mentioned during consultation, including in stressful situations, where it can be difficult to do so. Some participants suggested that recalling several times possible solutions could help patients acquire new reflexes. Nevertheless, some of them highlighted that proposing too many solutions, or using links that could inadvertently lead to symptoms or disorder-related unhelpful websites could jeopardize the commitment to the app. Patients said that their search history, impacted by previous searches, may accentuate these risks.

##### Theme: information

For both groups, the app’s feature concerning disorder-related information was one of the most important. Several patients said they had trouble looking for information about their disorder, having faced diverse and unclear information, or even incorrect information that could lead to false beliefs. Some patients said that internet or social media browsing about their disorder could accentuate food obsessions or their sense of guilt. For some patients this feature’s advantage was to access reliable information. Clinicians saw the opportunity to give information prior to the first consultation or during follow-up, hoping to facilitate the discussion. The groups expected the app to address different themes. Patients looked for medically reliable information about food and dietetics, and information that went “beyond food symptoms”, such as items concerning self-esteem. Clinicians expected disorder-related data, information about malnutrition’s physical consequences and items helping patients find out where they stand in their in symptoms’ evolution. Finally, both groups said they expected to find data helping patients face the loss of dietary cues and offering social skills and assertiveness strategies. The issue of giving parents information was raised solely by practitioners. They were divided between the advantages of letting parents access the app and the ones of leaving the patient in charge of sharing, or not, pieces of information they had learned. Some clinicians feared patients could feel invaded in their privacy and thus lose the upside of the app’s personal dimension. Others worried that too much information could induce confusion, and that patients might use this kind of information to hide their symptoms.

##### Theme: motivational content

Participants also expected a lot from the motivational content. Patients expected the app to collect personal information such as personality traits, personal values, objectives and life projects that could be modifiable through time. They also expected the app to intervene directly, either by “reminding” patients of this personal motivating content, or in sharing encouraging messages, quotes or media.

Participants also expected to be able to define objectives. They were perceived as motivation to change in themselves, by the rewards they could bring about, and by highlighting benefits changes could provoke. Whereas patients said they expected the app to generate challenges, that could be personalized and changeable, clinicians insisted that it would more relevant that these challenges be presented by the patients themselves. Several participants said the objectives should be short-termed and centered around symptoms or the openness towards others and activities. Patients suggested that long-term objectives, such as life plans, could be motivating. For both groups, recurrent and user-adjustable notifications about the objectives’ evolution could be useful. Some patients insisted nonetheless that these challenges should not produce any kind of pressure, whether in their formulation, their follow-up or in the rewards they can entail.

#### Use

##### Theme: relations with care

**Fig. 5:**
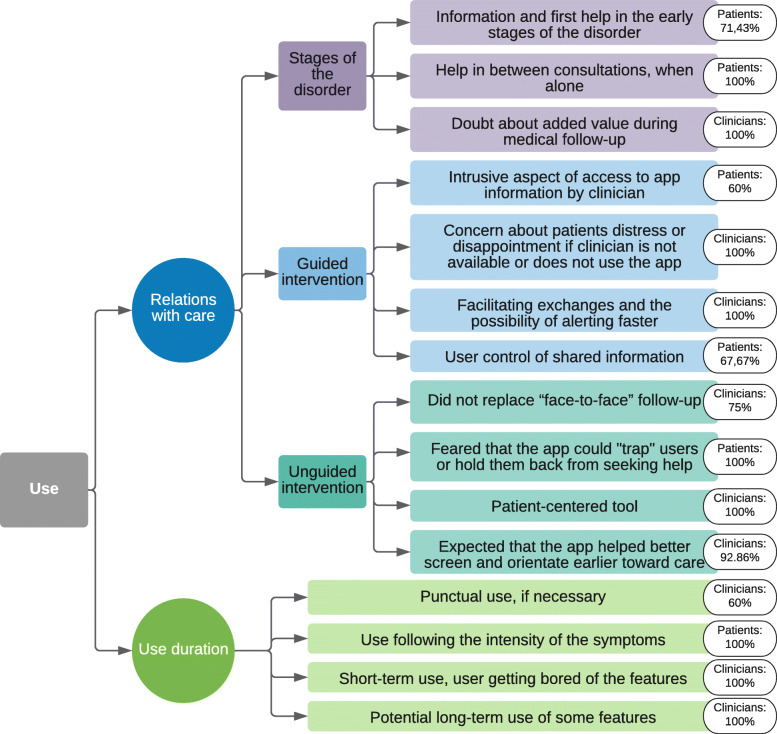
“Use” axis thematic arborescence describing main themes and subthemes with percentage of the highest group’s coding rate per theme

Participants expressed mixed opinions about how the app should be involved in patient care (Fig. 5). Some thought it would be beneficial that the app be linked to a clinician, by facilitating exchanges and the possibility of alerting faster when there is trouble. Others feared this kind of assistance could be intrusive as patient may use the app in a personal way and feared the loss of such privacy. Moreover, some clinicians imagined patients might expect practitioners to use this app and be available through it, thus creating hardships for patients anxiously waiting if answers are not provided. Patients expected to have the opportunity to talk about app use during consultation times, while keeping the amount of information they wished to share under control. The unguided nature of this app could allow this tool to be patient-centered, according to the clinician. It was expected that it could help screening patients early, and referring them towards proper care while encouraging them to play an active role in their treatment, by giving them contacts of suitable establishments (i.e., primary care workers, assistance help line, associations, dedicated facilities). Thus, it was necessary for clinicians to remind patients as soon as users installed the app that it was not a substitute for appropriate clinician-provided care. Patients bore in mind that this tool did not replace a “face-to-face” follow-up, even though some feared having this app would hold them back from asking for help by keeping them trapped in the app. For participants from both groups this app was seen as a tool essentially made for patients in the early stages of the disorder, that could provide initial assistance when treatment is not yet implemented. Some clinicians doubted it would offer added value in an ongoing follow-up. Conversely, one patient reported that it could be helpful between consultations.

##### Theme: use duration

Participants had different opinions concerning the potential time spent using the app. Some clinicians said it would probably be used sparsely, with the possibility of getting bored of the features. For other clinicians, some features like emotions’ evaluation and disorder-related information, if conveyed gradually, could be used for longer. Finally, clinicians thought the app could be used every now and then, according to the patient’s needs. Patients imagined using it occasionally, when they needed information, help or motivation. For them, this app’s use was not thought as consistent over a specific time-period, but dependent on the disorder’s evolution, and may diminish when the symptoms do.

## Discussion

This study focused on an essential step in app development by evaluating its early acceptability and user experience in its initial phase. In general, the app’s overall attractiveness was adequate, but the UEQ results showed weaker dimensions in user experience, including novelty, dependability, and efficiency, which matched some participants’ comments. Efficiency score was low on the UEQ, which participants confirmed by expressing mixed beliefs. Qualitative data showed that when participants thought the app was effective, it promoted its adoption, whereas it hindered it when they thought it was ineffective, which is consistent with current literature [28, 30]. Moreover, several models about apps adoption highlight that the perceived efficiency of a product is one of the key elements guiding the intent to use, which is essential in deciding whether or not to adopt a product [32, 33]. Participants’ expectations included that the app needed to be properly broadcasted (online and on different apps stores), and recommended by health workers. Indeed, Kim et al. [33] in their multi-stage adoption model state that social influence (i.e., how strongly an individual believes that important others think he or she should use the technology), which is also a key element in the intent to use, could influence positively the perceived efficiency. Spreading the app’s use could thus improve its credibility and expected efficiency [28]. Besides, it seems that being recommended by a health worker is the best way to boost an app’s adoption [63]. Concerning novelty, patients did not find the aesthetic very modern or appealing, which hindered the installation of the app. Clinicians, in contrast, were satisfied with the aesthetic. The low dependability score could be related to difficulties experienced by clinicians when navigating in the app, as well as patients’ opinions about design that fell short of their expectations. The app’s aesthetics and design are also key elements in its adoption process, still according to some comprehensive models of apps adoption. Indeed, on one hand, they take part in the user experience which contributes to the satisfaction during use, turning the intent to use into adoption [31–34]. On the other hand, aesthetics and design also seem to guide users as early as the app’s search or exploration, through screenshots displayed on stores, influencing the decision to install the app [31]. The study’s qualitative data shows the different expectations, towards these two dimensions, between adolescent users and adult clinicians. This highlights the importance of including users in the app development process as early as possible to better understand their needs and preferences [29, 38, 55]. This is particularly true for adolescents, who have always known these tools and use them daily. They will express different, often higher, expectations than adults, specifically about customization features [15, 30].

The qualitative data revealed many expectations for this application. Patients and clinicians agreed on the importance of the psychoeducation feature. Patients highlighted the difficulties they faced when they looked for information about the disorder, such as too much information, guilt-inducing information or even false information that could reinforce false beliefs or symptoms. These experiences are consistent with the Arts et al. Study that showed that AN online information was often of variable quality and difficult to read [64]. Even more, and especially for adolescents, not understanding the disorder and the need for treatment is a real obstacle in implementing appropriate treatment [9]. It was therefore expected that having a single source of reliable data on the disorder or on dietary facts would limit this risk and facilitate communication with caregivers. Patients also expected to be able to choose items about self-esteem and social skills, so the app would be relevant during disorder’s later stages. The user’s ability to choose elements that represent situations with which they identify seems, according to other studies [29, 30, 34, 55], to be a personalization feature that improves engagement and adherence.

Participants have also brought forward that there are internal factors, specific to the disorder, that could hinder the app’s adoption. Patients thus highlighted that shame and denial could be limiting factors. Feelings of shame were comprised of two dimensions. On one hand, they said that exposing oneself on account of owning an app dedicated to ED could be embarrassing through the eyes of others, for example because of untimely notifications, or the phone being looked “over” the shoulder. On the other hand, shame could be induced by reminding patients of their disorders, of the difficulties they face, reinforcing the distorted way they see themselves, which is an essential part of the disorder. Social influence, as was discussed earlier, is a major parameter in building the intent to use [32, 33]. It can therefore be influenced negatively by shame and disorder-related stigmas, which are well-known barriers to the search for help and treatment in EDs [8, 9], and more generally in mental health care. We can thus see how important it would be that the app felt safe and “without judgement”. Beyond these considerations, the app’s design allowing it to not being instantly labelled as a mental health app dedicated to EDs could improve the sense of security and intimacy patients are looking for and avoid self-stigmatization reinforcement. Choosing carefully the app’s title could also be important. For example, Huang et al. [65] who studied apps aiming to alleviate anxiety found that apps with a title referring directly to anxiety disorders or symptoms had a lower installation rate than those of which the title was not symptoms-related.

Gamification features were well accepted and could even be perceived as motivating and enjoyable. However, some participants expressed worries about some elements that could bring about obsessions or pressure. This underscores the importance of carefully choosing gamification mechanisms and how they are implemented to better meet patients’ needs and symptoms, avoiding adverse effects [52, 53, 66]. However, when properly implemented, these features can be great assets, still underused, in improving interventions’ efficiency and commitment to [13, 54, 55].

There were different points of view between clinicians and patients about the app’s use. Clinicians envisioned a short-term use, mainly in searching for help surrounding the disorder. They mostly expected the app to help screen patients and direct them towards proper care. Besides, it seems like short-term interventions are usually more efficient, maybe because apps tend to be less used over time [13]. Finally, some clinicians did not think this app would be useful in a “face-to-face” setting, or thought it could increase patients’ expectations, thus creating the risk of disappointment. Lindgreen et al. [67] showed that using this type of tool during consultations could also create distress for clinicians and lower their work satisfaction. Indeed, in this study, clinicians felt like they might not have the necessary technological skills, or that there could be a gap between patients’ expectations and theirs, thus potentially damaging the therapeutic relationship or alliance and diminishing patients’ trust in their clinicians. This shows how important it is to think about objectives and use recommendations for patients but also for clinicians, to avoid adding a burden to their practice. Patients seemed to expect a strong sense of intimacy with the app. Most imagined using it occasionally, if necessary, but expected a follow-up throughout the disorder’s evolution. They could thus come look for information or personal motivations (i.e., life projects, personal values) depending on their needs at that precise moment. Patients expressed needing more than text coping strategies, such as links to different media via the app. This concurs with Barakat’s et al. [17] results, stating that using several media types was associated with a better ED symptoms improvement. Furthermore, it seems that, during the pandemic, difficulties in regulating one’s emotions that ED patients face could have played a major part in ED symptoms aggravation in that period [10]. COVID-19’s impact on health care systems also shows the importance of developing new methods in helping ED patients when health care becomes less accessible [10, 11]. Finding efficient ways to distribute these coping strategies, thanks to the different medias they can provide, seems to be an e-health interventions’ relevant challenge.

This study’s main limitation was the selection bias: only hospitalized patients were recruited. This limits the results’ generalization to different stages of the disorder but, nonetheless, offers information about users’ needs at the start of the disorder and in already coordinated care. A second limitation was the small number of participants, so these results cannot be considered definitive or stable because we did not reach data saturation. However, we did not aim to be exhaustive, but rather to get an overview of important topics to address when developing an app, keeping in mind the flexibility a fast implementation needs. Many studies have insisted on the importance of using alternative methods in e-health intervention evaluation [15, 36, 38, 55]. Indeed, traditional evaluation methods, such as randomized controlled trials, are not compatible with development process, because of their long implementation timeline and the fast-evolving users’ expectations and technological progresses. Rapid and iterative testing methods with results implementation involving users in their development allow this flexibility. Tools used during this study are an example of these kinds of tests. More than allowing to punctually evaluate the app, they can assist in building a reference to better monitor different versions’ evolution and improvement features that must be implemented over time [36]. Traditional methods are still relevant in evaluating sparse data, like efficiency, once this process is over [15].

## Conclusion

Developing new technologies in AN accompaniment could enable to bypass some of the barriers they face when trying to access specific care. These solutions are not however usually very engaging for users, particularly in adolescent population which have different expectations than adults. This study shows the importance of including users in the different stages of an e-health intervention development. It identifies needs, general patterns of use and adherence, which may be very different from those envisioned by clinicians. Patients’ expectations were mainly centered around getting reliable information as well as an emotional and motivational accompaniment. Clinicians mostly expected the app to help health workers screen and evaluate patients, rather than for follow-up. Several barriers to the app’s adoption were outlined, especially by the patients, such as design and aesthetics, or doubt in these tools’ efficiency. However, suggestions for improving the adoption of the application were made, like internet broadcasting and professional recommendation, and making the app design non-stigmatizing. These findings could help to make the right changes in order to meet users’ needs, which could significantly improve the program’s adoption and adherence, and thus its effectiveness. Developing a mobile app is a continuous process that should regularly include these evaluations, until its efficiency is properly evaluated.

## Data Availability

The dataset supporting the findings of this study is available from the corresponding author, BN, upon reasonable request. The data are not publicly available due to legal and ethical restrictions.

## Abbreviations

AN: Anorexia Nervosa
CBT: Cognitive-Behavioral Therapy
CI: Confidence Interval
ED: Eating Disorders
UEQ: User Experience Questionnaire

## References

[1] Keshaviah A, Edkins K, Hastings ER, Krishna M, Franko DL, Herzog DB, Thomas JJ, Murray HB, Eddy KT (2014). Re-examining premature mortality in anorexia nervosa: a meta-analysis redux. Compr Psychiatry.

[2] Fichter MM, Quadflieg N (2016). Mortality in eating disorders - results of a large prospective clinical longitudinal study. Int J Eat Disord.

[3] Chesney E, Goodwin GM, Fazel S (2014). Risks of all-cause and suicide mortality in mental disorders: a meta-review. World Psychiatry Off J World Psychiatr Assoc WPA.

[4] Roux H, Chapelon E, Godart N (2013). Epidemiology of anorexia nervosa: a review. L’Encephale.

[5] Pollack LO, McCune AM, Mandal K, Lundgren JD. Quantitative and qualitative analysis of the quality of life of individuals with eating disorders. Prim Care Companion CNS Disord. 2015;17. 10.4088/PCC.14m01667. 10.4088/PCC.14m01667 PMC4560188 26445689

[6] Zipfel S, Löwe B, Reas DL, Deter H-C, Herzog W. Long-term prognosis in anorexia nervosa: lessons from a 21-year follow-up study. Lancet 2000;355:721–722. 10.1016/S0140-6736(99)05363-5, 9205. 10.1016/S0140-6736(99)05363-5 10703806

[7] Zipfel S, Giel KE, Bulik CM, Hay P, Schmidt U. Anorexia nervosa: aetiology, assessment, and treatment. Lancet Psychiatry 2015;2:1099–1111. 10.1016/S2215-0366(15)00356-9, 12. 10.1016/S2215-0366(15)00356-9 26514083

[8] Johns G, Taylor B, John A, Tan J (2019). Current eating disorder healthcare services – the perspectives and experiences of individuals with eating disorders, their families and health professionals: systematic review and thematic synthesis. BJPsych Open.

[9] Kästner D, Weigel A, Buchholz I, Voderholzer U, Löwe B, Gumz A (2021). Facilitators and barriers in anorexia nervosa treatment initiation: a qualitative study on the perspectives of patients, carers and professionals. J Eat Disord.

[10] Vuillier L, May L, Greville-Harris M, Surman R, Moseley RL (2021). The impact of the COVID-19 pandemic on individuals with eating disorders: the role of emotion regulation and exploration of online treatment experiences. J Eat Disord.

[11] Richardson C, Patton M, Phillips S, Paslakis G (2020). The impact of the COVID-19 pandemic on help-seeking behaviors in individuals suffering from eating disorders and their caregivers. Gen Hosp Psychiatry.

[12] Revet A, Hebebrand J, Anagnostopoulos D, Kehoe LA, Gradl-Dietsch G, COVID-19 Child and Adolescent Psychiatry Consortium, et al. Perceived impact of the COVID-19 pandemic on child and adolescent psychiatric services after 1 year (February/March 2021): ESCAP CovCAP survey. Eur Child Adolesc Psychiatry. 2021. 10.1007/s00787-021-01851-1. 10.1007/s00787-021-01851-1 PMC8318839 34322720

[13] Iribarren SJ, Akande TO, Kamp KJ, Barry D, Kader YG, Suelzer E (2021). Effectiveness of Mobile apps to promote health and manage disease: systematic review and Meta-analysis of randomized controlled trials. JMIR MHealth UHealth.

[14] Loo Gee B, Griffiths KM, Gulliver A (2016). Effectiveness of mobile technologies delivering ecological momentary interventions for stress and anxiety: a systematic review. J Am Med Inform Assoc JAMIA.

[15] Scholten H, Granic I (2019). Use of the principles of design thinking to address limitations of digital mental health interventions for youth: viewpoint. J Med Internet Res.

[16] Torok M, Han J, Baker S, Werner-Seidler A, Wong I, Larsen M (2019). Suicide prevention using self-guided digital interventions: a systematic review and meta-analysis of randomised controlled trials. Lancet Digit Health.

[17] Barakat S, Maguire S, Smith KE, Mason TB, Crosby RD, Touyz S (2019). Evaluating the role of digital intervention design in treatment outcomes and adherence to eTherapy programs for eating disorders: a systematic review and meta-analysis. Int J Eat Disord.

[18] Hötzel K, von Brachel R, Schmidt U, Rieger E, Kosfelder J, Hechler T, Schulte D, Vocks S (2014). An internet-based program to enhance motivation to change in females with symptoms of an eating disorder: a randomized controlled trial. Psychol Med.

[19] Cardi V, Albano G, Ambwani S, Cao L, Crosby RD, Macdonald P, Schmidt U, Treasure J (2019). A randomised clinical trial to evaluate the acceptability and efficacy of an early phase, online, guided augmentation of outpatient care for adults with anorexia nervosa. Psychol Med.

[20] Brewin N, Wales J, Cashmore R, Plateau CR, Dean B, Cousins T, Arcelus J (2016). Evaluation of a motivation and psycho-educational guided self-help intervention for people with eating disorders (MOPED). Eur Eat Disord Rev J Eat Disord Assoc.

[21] Aardoom JJ, Dingemans AE, van Ginkel JR, Spinhoven P, Van Furth EF, Van den Akker-van Marle ME (2016). Cost-utility of an internet-based intervention with or without therapist support in comparison with a waiting list for individuals with eating disorder symptoms: a randomized controlled trial. Int J Eat Disord.

[22] Aardoom JJ, Dingemans AE, Van Furth EF (2016). E-health interventions for eating disorders: emerging findings, issues, and opportunities. Curr Psychiatry Rep.

[23] Fairburn CG, Rothwell ER (2015). Apps and eating disorders: a systematic clinical appraisal. Int J Eat Disord.

[24] Juarascio AS, Manasse SM, Goldstein SP, Forman EM, Butryn ML (2015). Review of smartphone applications for the treatment of eating disorders. Eur Eat Disord Rev.

[25] Ahmadiankalati M, Steins-Loeber S, Paslakis G. Review of randomized controlled trials using e-health interventions for patients with eating disorders. Front Psychiatry. 2020;11. 10.3389/fpsyt.2020.00568. 10.3389/fpsyt.2020.00568 PMC7304304 32595546

[26] Fry JP, Neff RA (2009). Periodic prompts and reminders in health promotion and health behavior interventions: systematic review. J Med Internet Res.

[27] Webb TL, Joseph J, Yardley L, Michie S (2010). Using the internet to promote health behavior change: a systematic review and meta-analysis of the impact of theoretical basis, use of behavior change techniques, and mode of delivery on efficacy. J Med Internet Res.

[28] Beatty L, Binnion C (2016). A systematic review of predictors of, and reasons for, adherence to online psychological interventions. Int J Behav Med.

[29] Ludden GDS, van Rompay TJL, Kelders SM, van Gemert-Pijnen JEWC (2015). How to increase reach and adherence of web-based interventions: a design research viewpoint. J Med Internet Res.

[30] Achilles MR, Anderson M, Li SH, Subotic-Kerry M, Parker B, O’Dea B (2020). Adherence to e-mental health among youth: considerations for intervention development and research design. Digit Health.

[31] Schueller SM, Neary M, O’Loughlin K, Adkins EC (2018). Discovery of and interest in health apps among those with mental health needs: survey and focus group study. J Med Internet Res.

[32] Malik A, Suresh S, Sharma S (2017). Factors influencing consumers’ attitude towards adoption and continuous use of mobile applications: a conceptual model. Procedia Comput Sci.

[33] Kim A, Kim K (2014). User experience and the multi-stage adoption of Mobile apps. J Inf Technol Appl Manag.

[34] Lallemand C, Gronier G. Méthodes de design UX: 30 méthodes fondamentales pour concevoir des expériences optimales. 2nd ed. Eyrolles; 2018.

[35] Murray E. Web-Based Interventions for Behavior Change and Self-Management: Potential, Pitfalls, and Progress. Med 20 2012;1:e3. 10.2196/med20.1741. 10.2196/med20.1741 PMC4084772 25075231

[36] Nitsch M, Dimopoulos CN, Flaschberger E, Saffran K, Kruger JF, Garlock L, Wilfley DE, Taylor CB, Jones M (2016). A guided online and Mobile self-help program for individuals with eating disorders: an iterative engagement and usability study. J Med Internet Res.

[37] Schlegl S, Bürger C, Schmidt L, Herbst N, Voderholzer U (2015). The potential of technology-based psychological interventions for anorexia and bulimia nervosa: a systematic review and recommendations for future research. J Med Internet Res.

[38] Molina-Recio G, Molina-Luque R, Jiménez-García AM, Ventura-Puertos PE, Hernández-Reyes A, Romero-Saldaña M (2020). Proposal for the user-centered design approach for health apps based on successful experiences: integrative review. JMIR MHealth UHealth.

[39] Ambwani S, Cardi V, Treasure J (2014). Mobile self-help interventions for anorexia nervosa: conceptual, ethical, and methodological considerations for clinicians and researchers. Prof Psychol Res Pract.

[40] Yim SH, Schmidt U (2019). Self-help treatment of eating disorders. Psychiatr Clin North Am.

[41] Denison-Day J, Appleton KM, Newell C, Muir S (2018). Improving motivation to change amongst individuals with eating disorders: a systematic review. Int J Eat Disord.

[42] Haynos AF, Fruzzetti AE (2011). Anorexia nervosa as a disorder of emotion dysregulation: evidence and treatment implications. Clin Psychol Sci Pract.

[43] Lavender JM, Wonderlich SA, Engel SG, Gordon KH, Kaye WH, Mitchell JE (2015). Dimensions of emotion dysregulation in anorexia nervosa and bulimia nervosa: a conceptual review of the empirical literature. Clin Psychol Rev.

[44] Prefit A-B, Cândea DM, Szentagotai-Tătar A (2019). Emotion regulation across eating pathology: a meta-analysis. Appetite.

[45] Selby EA, Cornelius T, Fehling KB, Kranzler A, Panza EA, Lavender JM, Wonderlich SA, Crosby RD, Engel SG, Mitchell JE, Crow SJ, Peterson CB, Grange DL (2015). A perfect storm: examining the synergistic effects of negative and positive emotional instability on promoting weight loss activities in anorexia nervosa. Front Psychol.

[46] Dalle Grave R, Eckhardt S, Calugi S, Le Grange D (2019). A conceptual comparison of family-based treatment and enhanced cognitive behavior therapy in the treatment of adolescents with eating disorders. J Eat Disord.

[47] Dalle Grave R, El Ghoch M, Sartirana M, Calugi S (2016). Cognitive behavioral therapy for anorexia nervosa: an update. Curr Psychiatry Rep.

[48] Lynch TR, Gray KL, Hempel RJ, Titley M, Chen EY, O’Mahen HA (2013). Radically open-dialectical behavior therapy for adult anorexia nervosa: feasibility and outcomes from an inpatient program. BMC Psychiatry.

[49] Chen EY, Segal K, Weissman J, Zeffiro TA, Gallop R, Linehan MM, Bohus M, Lynch TR (2015). Adapting dialectical behavior therapy for outpatient adult anorexia nervosa--a pilot study. Int J Eat Disord.

[50] Watson D, Clark LA, Tellegen A (1988). Development and validation of brief measures of positive and negative affect: the PANAS scales. J Pers Soc Psychol.

[51] Caci H, Baylé J. l’échelle d’affectivité positive et d’affectivité négative. Première traduction en français. Congrès L’Encéphale Paris 2007.

[52] Sardi L, Idri A, Fernández-Alemán JL (2017). A systematic review of gamification in e-health. J Biomed Inform.

[53] Cheng VWS, Davenport T, Johnson D, Vella K, Hickie IB (2019). Gamification in apps and Technologies for Improving Mental Health and Well-Being: systematic review. JMIR Ment Health.

[54] Hamari J, Koivisto J, Sarsa H. Does Gamification Work? – A Literature Review of Empirical Studies on Gamification. 2014 47th Hawaii Int. Conf Syst Sci. 2014:3025–34. 10.1109/HICSS.2014.377.

[55] Fleming TM, de Beurs D, Khazaal Y, Gaggioli A, Riva G, Botella C, et al. Maximizing the impact of e-therapy and serious gaming: time for a paradigm shift. Front Psychiatry. 2016;7. 10.3389/fpsyt.2016.00065. 10.3389/fpsyt.2016.00065 PMC4834305 27148094

[56] Laugwitz B, Held T (2008). Schrepp M. Construction and Evaluation of a User Experience Questionnaire vol.

[57] Schrepp M, Hinderks A, Thomaschewski J (2017). Construction of a benchmark for the user experience questionnaire (UEQ). Int J Interact Multimed Artif Intell.

[58] Hassenzahl M (2005). The thing and I: understanding the relationship between user and product. Funology Usability Enjoyment.

[59] Thüring M, Mahlke S (2007). Usability, aesthetics and emotions in human–technology interaction. Int J Psychol.

[60] Martin N, Erhel S, Jamet É, Rouxel G. What links between user experience and acceptability? Proc. 27th Conf. Interact. Homme-Mach., Toulouse, France: Association for Computing Machinery; 2015, p. 1–6. 10.1145/2820619.2825015.

[61] Paillé P, Mucchielli A (2016). L’analyse qualitative en sciences humaines et sociales - 4e éd. 4e édition.

[62] Tong A, Sainsbury P, Craig J (2008). Consolidated criteria for reporting qualitative research (COREQ): a 32-item checklist for interviews and focus groups. Int J Qual Health Care J Int Soc Qual Health Care ISQua.

[63] Lipschitz J, Miller CJ, Hogan TP, Burdick KE, Lippin-Foster R, Simon SR, Burgess J (2019). Adoption of Mobile apps for depression and anxiety: cross-sectional survey study on patient interest and barriers to engagement. JMIR Ment Health.

[64] Arts H, Lemetyinen H, Edge D (2020). Readability and quality of online eating disorder information-are they sufficient? A systematic review evaluating websites on anorexia nervosa using DISCERN and Flesch readability. Int J Eat Disord.

[65] Huang H-Y, Bashir M (2017). Users’ adoption of mental health apps: examining the impact of information cues. JMIR MHealth UHealth.

[66] Schmidt-Kraepelin M, Toussaint PA, Thiebes S, Hamari J, Sunyaev A (2020). Archetypes of gamification: analysis of mHealth apps. JMIR MHealth UHealth.

[67] Lindgreen P, Clausen L, Lomborg K (2018). Clinicians’ perspective on an app for patient self-monitoring in eating disorder treatment. Int J Eat Disord.

